# Supplementary material for: Control of tissue development and cell diversity by cell cycle-dependent transcriptional filtering
Source: eLife. 2021 Jul 2;10:e64951. doi: 10.7554/eLife.64951 (PMC8279763; doi:10.7554/eLife.64951)
Supplement: Supplementary file 1. [file elife-64951-supp1.docx]

Table S1: Curated cell cycle duration data

| YEAR | PMID | SPECIES | TISSUE | Developmental  timepoint | T_C | unit |
| --- | --- | --- | --- | --- | --- | --- |
| 1962 | 13960119 | g. gallus | brain | E6 | 16 | hrs |
| 1962 | 13960119 | g. gallus | brain | E1 | 5 | hrs |
| 1965 | 5859018 | m. musculus | brain | E11 | 11 | hrs |
| 1959 | 13672199 | m. musculus | intestine |  | 9.5 | hrs |
| 1960 | 13738646 | m. musculus | intestine |  | 19 | hrs |
| 1960 | 13738646 | t. paludosa | root |  | 17.3 | hrs |
| 1962 | 14465693 | m. musculus | intestine | Adult | 16 | hrs |
| 1964 | 14105210 | g. gallus | epithelial | P2 | 11 | hrs |
| 1964 | 14105210 | m. musculus | Trophoblast | E12 | 15 | hrs |
| 1964 | 14105210 | m. musculus | Trophoblast | E8 | 9.4 | hrs |
| 1967 | 6058220 | rattus | retina | P2 | 28 | hrs |
| 1965 | 14268461 | m. musculus | jejunum |  | 18 | hrs |
| 1965 | 14268461 | m. musculus | tounge |  | 61 | hrs |
| 1965 | 14268461 | m. musculus | Esophagus |  | 87 | hrs |
| 1965 | 14268461 | m. musculus | Abdominal skin | | 151 | hrs |
| 1965 | 14268461 | m. musculus | forestomachs | | 168 | hrs |
| 1965 | 14268461 | m. musculus | liver |  | 173 | hrs |
| 1965 | 14268461 | m. musculus | kidney |  | 608 | hrs |
| 1968 | 5760443 | m. musculus | neural tube | E10 | 8.56 | hrs |
| 1968 | 5760443 | m. musculus | neural tube | E11 | 10.5 | hrs |
| 1971 | 5542640 | m. musculus | ectoderm | E7.5 | 6.25 | hrs |
| 1971 | 5542640 | m. musculus | mesoderm | E7.5 | 7.5 | hrs |
| 1971 | 5542640 | m. musculus | mesoderm | E7.5 | 8.6 | hrs |
| 1971 | 5542640 | m. musculus | ectoderm | E7.5 | 6.5 | hrs |
| 1972 | 4635365 | wistar rat | brain | E12 | 13.1 | hrs |
| 1972 | 4635365 | wistar rat | brain | E13 | 14.2 | hrs |
| 1972 | 4635365 | wistar rat | brain | E14 | 12.9 | hrs |
| 1972 | 4635365 | wistar rat | brain | E15 | 18.4 | hrs |
| 1972 | 4635365 | wistar rat | brain | E16 | 17 | hrs |
| 1972 | 4635365 | wistar rat | brain | E17 | 16.8 | hrs |
| 1972 | 4635365 | wistar rat | brain | E18 | 20.8 | hrs |
| 1972 | 4635365 | wistar rat | brain | E12 | 11.3 | hrs |
| 1972 | 4635365 | wistar rat | brain | E13 | 12.6 | hrs |
| 1972 | 4635365 | wistar rat | brain | E14 | 11.3 | hrs |
| 1972 | 4635365 | wistar rat | brain | E15 | 16.9 | hrs |
| 1972 | 4635365 | wistar rat | brain | E16 | 16.5 | hrs |
| 1972 | 4635365 | wistar rat | brain | E17 | 16.8 | hrs |
| 1972 | 4635365 | wistar rat | brain | E18 | 21.8 | hrs |
| 1972 | 4635365 | wistar rat | brain | E12 | 9.8 | hrs |
| 1972 | 4635365 | wistar rat | brain | E13 | 10.7 | hrs |
| 1972 | 4635365 | wistar rat | brain | E14 | 9.8 | hrs |
| 1972 | 4635365 | wistar rat | brain | E15 | 13.7 | hrs |
| 1972 | 4635365 | wistar rat | brain | E16 | 12.8 | hrs |
| 1972 | 4635365 | wistar rat | brain | E17 | 12.6 | hrs |
| 1972 | 4635365 | wistar rat | brain | E18 | 15.3 | hrs |
| 1975 | 114907 | rattus | intestine | P90 | 11.32 | hrs |
| 1975 | 114907 | rattus | intestine | P91 | 11 | hrs |
| 1976 | 1001817 | mouse | retina | E15 | 20 | hrs |
| 1983 | 6625191 | NMRI-mouse | lateral hemispheric wall | E12 | 10.02 | hrs |
| 1983 | 6625191 | NMRI-mouse | medial hemispheric wall | E12 | 10.23 | hrs |
| 1983 | 6625191 | NMRI-mouse | lateral hemispheric wall | E13 | 10.76 | hrs |
| 1983 | 6625191 | NMRI-mouse | medial hemispheric wall | E13 | 11.09 | hrs |
| 1983 | 6625191 | NMRI-mouse | medial hemispheric wall | E15 | 12.98 | hrs |
| 1983 | 6625191 | NMRI-mouse | lateral hemispheric wall | E15 | 15.48 | hrs |
| 1983 | 6625191 | NMRI-mouse | medial hemispheric wall | E17 | 15.38 | hrs |
| 1983 | 6625191 | NMRI-mouse | lateral hemispheric wall | E17 | 18.56 | hrs |
| 1983 | 6411748 | d. melanogaster | larvae | cell cycle 10 | 9 | min |
| 1983 | 6411748 | d. melanogaster | larvae | cell cycle 11 | 10 | min |
| 1983 | 6411748 | d. melanogaster | larvae | cell cycle 12 | 12 | min |
| 1983 | 6411748 | d. melanogaster | larvae | cell cycle 13 | 21 | min |
| 1985 | 4041905 | m. musculus | retina | E7 | 6.25 | hrs |
| 1985 | 4041905 | m. musculus | retina | E10 | 10 | hrs |
| 1985 | 4041905 | m. musculus | retina | E15 | 20 | hrs |
| 1985 | 4041905 | m. musculus | retina | P1 | 30 | hrs |
| 1989 | 2746304 | m. musculus | denate gyrus | P20 | 16.1 | hrs |
| 1989 | 2746304 | m. musculus | dentate gyrus | P20 | 16 | hrs |
| 1989 | 2746304 | m. musculus | dentate gyrus | P20 | 9.8 | hrs |
| 1995 | 7666189 | m. musculus | cerebral wall | spp | 15.1 | hrs |
| 1995 | 7666189 | m. musculus | cerebral wall | pve | 15 | hrs |
| 1995 | 7666189 | m. musculus | cerebral wall | spp | 17.5 | hrs |
| 1995 | 7666189 | m. musculus | cerebral wall | pve | 15 | hrs |
| 1995 | 7666189 | m. musculus | cerebral wall | spp | 18.4 | hrs |
| 1995 | 7666189 | m. musculus | cerebral wall | pve | 15 | hrs |
| 1995 | 7666188 | m. musculus | cerebral wall | E11 | 8.1 | hrs |
| 1995 | 7666188 | m. musculus | cerebral wall | E12 | 10.2 | hrs |
| 1995 | 7666188 | m. musculus | cerebral wall | E13 | 11.4 | hrs |
| 1995 | 7666188 | m. musculus | cerebral wall | E14 | 15.1 | hrs |
| 1995 | 7666188 | m. musculus | cerebral wall | E15 | 17.5 | hrs |
| 1995 | 7666188 | m. musculus | cerebral wall | E16 | 18.4 | hrs |
| 1998 | 9448316 | m. mulatta | brain | E40 | 22.7 | hrs |
| 1998 | 9448316 | m. mulatta | brain | E60 | 54.1 | hrs |
| 1998 | 9448316 | m. mulatta | brain | E80 | 27.4 | hrs |
| 2002 | 12151540 | m. musculus | cortical stem cell | E14 | 29.4 | hrs |
| 2002 | 12151540 | m. musculus | cortical stem cell | E14 | 27.6 | hrs |
| 2002 | 12151540 | m. musculus | cortical stem cell | E15 | 29.3 | hrs |
| 2002 | 12151540 | m. musculus | cortical stem cell | E15 | 24.4 | hrs |
| 2002 | 12151540 | m. musculus | cortical stem cell | E15 | 28.4 | hrs |
| 2002 | 12151540 | m. musculus | cortical stem cell | E15 | 25.5 | hrs |
| 2002 | 12151540 | m. musculus | cortical stem cell | E16 | 30 | hrs |
| 2002 | 12151540 | m. musculus | cortical stem cell | E16 | 27.7 | hrs |
| 2008 | 18164540 | m. musculus | embryo | E1 | 19 | hrs |
| 2008 | 18164540 | m. musculus | embryo | E2 | 20 | hrs |
| 2008 | 18164540 | m. musculus | embryo | E3 | 11 | hrs |
| 2008 | 18164540 | m. musculus | embryo | E4 | 11 | hrs |
| 2008 | 18430415 | c. elegans | Msa | L | 69.8 | min |
| 2008 | 18430415 | c. elegans | MSp | R | 69.9 | min |
| 2008 | 18430415 | c. elegans | Mspa | R | 94.5 | min |
| 2008 | 18430415 | c. elegans | Msaa | L | 94.6 | min |
| 2008 | 18430415 | c. elegans | Msap | L | 96.6 | min |
| 2008 | 18430415 | c. elegans | MSpp | R | 96.7 | min |
| 2008 | 18430415 | c. elegans | Msaaa | L | 123.2 | min |
| 2008 | 18430415 | c. elegans | Mspap | R | 123.5 | min |
| 2008 | 18430415 | c. elegans | Msaap | L | 123.8 | min |
| 2008 | 18430415 | c. elegans | Mspaa | R | 124.1 | min |
| 2008 | 18430415 | c. elegans | Msapa | L | 126.8 | min |
| 2008 | 18430415 | c. elegans | Msppa | R | 126.9 | min |
| 2008 | 18430415 | c. elegans | Msppp | R | 127.2 | min |
| 2008 | 18430415 | c. elegans | Msapp | L | 127.5 | min |
| 2008 | 18430415 | c. elegans | Ear | R | 131.9 | min |
| 2008 | 18430415 | c. elegans | Eal | L | 132.1 | min |
| 2008 | 18430415 | c. elegans | Da | L | 134.7 | min |
| 2008 | 18430415 | c. elegans | Epr | R | 135.2 | min |
| 2008 | 18430415 | c. elegans | Epl | L | 135.4 | min |
| 2008 | 18430415 | c. elegans | Dp | R | 135.6 | min |
| 2008 | 18430415 | c. elegans | Msaaaa | L | 154.8 | min |
| 2008 | 18430415 | c. elegans | Mspaap | R | 155.3 | min |
| 2008 | 18430415 | c. elegans | Mspapa | R | 155.3 | min |
| 2008 | 18430415 | c. elegans | Msaaap | L | 155.4 | min |
| 2008 | 18430415 | c. elegans | Msaapa | L | 155.4 | min |
| 2008 | 18430415 | c. elegans | Mspaaa | R | 155.6 | min |
| 2008 | 18430415 | c. elegans | Msppap | R | 162.7 | min |
| 2008 | 18430415 | c. elegans | Mspapp | R | 162.8 | min |
| 2008 | 18430415 | c. elegans | Msapap | L | 162.9 | min |
| 2008 | 18430415 | c. elegans | Msaapp | L | 163 | min |
| 2008 | 18430415 | c. elegans | Msapaa | L | 163.7 | min |
| 2008 | 18430415 | c. elegans | Msppaa | R | 164 | min |
| 2008 | 18430415 | c. elegans | Mspppa | R | 166.7 | min |
| 2008 | 18430415 | c. elegans | Msappa | L | 169.4 | min |
| 2008 | 18430415 | c. elegans | Mspppp | R | 169.4 | min |
| 2008 | 18430415 | c. elegans | Msappp | L | 171.2 | min |
| 2008 | 18430415 | c. elegans | Dap | L | 175.2 | min |
| 2008 | 18430415 | c. elegans | Dpp | R | 175.6 | min |
| 2008 | 18430415 | c. elegans | Daa | L | 180.3 | min |
| 2008 | 18430415 | c. elegans | Dpa | R | 180.4 | min |
| 2013 | 24139044 | m. fascicularis | Ventricular zone | E48 | 43.5 | hrs |
| 2013 | 24139044 | m. fascicularis | Ventricular zone | E65 | 63 | hrs |
| 2013 | 24139044 | m. fascicularis | Outer SubVentricular zone | E65 | 71 | hrs |
| 2013 | 24139044 | m. fascicularis | ventricular zone | E78 | 45 | hrs |
| 2013 | 24139044 | m. fascicularis | outer SubVentricular zone | E78 | 47.5 | hrs |
