# Supplementary material for: Control of tissue development and cell diversity by cell cycle-dependent transcriptional filtering
Source: eLife. 2021 Jul 2;10:e64951. doi: 10.7554/eLife.64951 (PMC8279763; doi:10.7554/eLife.64951)
Supplement: Supplementary file 3. — The length of each gene was calculated using start and end positions for each gene as extracted from the Ensembl genome database (version 95). Estimated developmental time was curated from the Encyclopedia of Life or articles found in PubMed. [file elife-64951-supp3.docx]

Table S3: Curated developmental time for species (Ensembl version 95) and their corresponding median gene length

| Genome data source | (Developmental time)Days | Class | Order | Cell-Types_Vogel-valentine | Median gene length |
| --- | --- | --- | --- | --- | --- |
| acarolinensis_gene_ensembl | 27 | Reptilia | Squamata | 125 | 16406.5 |
| amelanoleuca_gene_ensembl | 120 | Mammalia | Ursidae | 145 | 14032.5 |
| amexicanus_gene_ensembl | 1 | Actinopterygii | Characiformes | 135 | 11511.5 |
| anancymaae_gene_ensembl | 133 | Mammalia | Primates | 215 | 28504 |
| aplatyrhynchos_gene_ensembl | 30 | Aves | Anseriformes | 175 | 9141 |
| btaurus_gene_ensembl | 281 | Mammalia | Artiodactyla | 200 | 15283.5 |
| caperea_gene_ensembl | 68 | Mammalia | Rodentia | 145 | 14649 |
| catys_gene_ensembl | 165 | Mammalia | Primates | 215 | 32312 |
| ccapucinus_gene_ensembl | 167.5 | Mammalia | Primates | 215 | 29218 |
| cchok1gshd_gene_ensembl | 16 | Mammalia | Rodentia | 145 | 17511 |
| ccrigri_gene_ensembl | 16 | Mammalia | Rodentia | 145 | 18414 |
| celegans_gene_ensembl | 0.5 | Chromadorea | Rhabditida | 16 | 2679 |
| cfamiliaris_gene_ensembl | 63 | Mammalia | Carnivora | 200 | 19941 |
| chircus_gene_ensembl | 150 | Mammalia | Artiodactyla | 200 | 21632 |
| choffmanni_gene_ensembl | 259 | Mammalia | Pilosa | 200 | 14475 |
| cintestinalis_gene_ensembl | 0.729 | Ascidiacea | Enterogona | 38 | 2620.5 |
| cjacchus_gene_ensembl | 150 | Mammalia | Primates | 215 | 31177 |
| clanigera_gene_ensembl | 111 | Mammalia | Rodentia | 145 | 25637 |
| cpalliatus_gene_ensembl | 162.5 | Mammalia | Primates | 215 | 28864 |
| cporcellus_gene_ensembl | 68 | Mammalia | Rodentia | 145 | 22711 |
| csabaeus_gene_ensembl | 165 | Mammalia | Primates | 215 | 17857 |
| csavignyi_gene_ensembl | 0.5 | Ascidiacea | Enterogona | 38 | 6542 |
| csyrichta_gene_ensembl | 182 | Mammalia | Primates | 215 | 22682 |
| dmelanogaster_gene_ensembl | 1 | Insecta | Diptera | 90 | 4166 |
| dnovemcinctus_gene_ensembl | 120 | Mammalia | Cingulata | 200 | 14420 |
| dordii_gene_ensembl | 29 | Mammalia | Rodentia | 145 | 19923 |
| drerio_gene_ensembl | 3 | Actinopterygii | Cypiniformes | 135 | 18232 |
| eburgeri_gene_ensembl | 150 | Myxini | Myxiniformes | 125 | 64084.5 |
| ecaballus_gene_ensembl | 338 | Mammalia | Perissodactyla | 200 | 13681.5 |
| eeuropaeus_gene_ensembl | 35 | Mammalia | Eulipotyphla | 200 | 17537 |
| etelfairi_gene_ensembl | 63 | Mammalia | Afrosoricida | 200 | 15604 |
| falbicollis_gene_ensembl | 16 | Aves | Passeriformes | 175 | 12637 |
| fcatus_gene_ensembl | 65 | Mammalia | Carnivora | 200 | 27833 |
| fdamarensis_gene_ensembl | 84 | Mammalia | Rodentia | 145 | 20308 |
| gaculeatus_gene_ensembl | 8 | Actinopterygii | Gastrosteiformes | 135 | 5143.5 |
| ggallus_gene_ensembl | 21 | Aves | Galliformes | 175 | 11342 |
| ggorilla_gene_ensembl | 270 | Mammalia | Primates | 215 | 28162 |
| gmorhua_gene_ensembl | 21 | Actinopterygii | Gadiformes | 135 | 6338.5 |
| hfemale_gene_ensembl | 70 | Mammalia | Rodentia | 145 | 19813 |
| hmale_gene_ensembl | 70 | Mammalia | Rodentia | 145 | 17269 |
| hsapiens_gene_ensembl | 274 | Mammalia | Primates | 215 | 34984 |
| itridecemlineatus_gene_ensembl | 40 | Mammalia | Rodentia | 145 | 22244.5 |
| jjaculus_gene_ensembl | 34 | Mammalia | Rodentia | 145 | 21692 |
| lafricana_gene_ensembl | 670 | Mammalia | Proboscidea | 200 | 16007 |
| lchalumnae_gene_ensembl | 420 | Sarcopterygii | ‎Coelacanthiformes | 150 | 23870 |
| loculatus_gene_ensembl | 7 | Actinopterygii | Semionotiformes | 135 | 13281 |
| mauratus_gene_ensembl | 18 | Mammalia | Rodentia | 145 | 19634 |
| mcaroli_gene_ensembl | 18.1 | Mammalia | Rodentia | 145 | 27105 |
| mdomestica_gene_ensembl | 14 | Mammalia | Didelphimorphia | 200 | 19506.5 |
| mfascicularis_gene_ensembl | 165 | Mammalia | Primates | 215 | 32198 |
| mfuro_gene_ensembl | 42 | Mammalia | Carnivora | 200 | 15474 |
| mgallopavo_gene_ensembl | 28 | Aves | Galliformes | 175 | 11180 |
| mleucophaeus_gene_ensembl | 179 | Mammalia | Primates | 215 | 29223 |
| mlucifugus_gene_ensembl | 50 | Mammalia | Chiroptera | 200 | 9982 |
| mmulatta_gene_ensembl | 165 | Mammalia | Primates | 215 | 31518 |
| mmurinus_gene_ensembl | 60 | Mammalia | Primates | 215 | 29104 |
| mmusculus_gene_ensembl | 20 | Mammalia | Rodentia | 145 | 24248 |
| mnemestrina_gene_ensembl | 180 | Mammalia | Primates | 215 | 33812 |
| mochrogaster_gene_ensembl | 25 | Mammalia | Rodentia | 145 | 20498 |
| mpahari_gene_ensembl | 22 | Mammalia | Rodentia | 145 | 26937.5 |
| mspretus_gene_ensembl | 20 | Mammalia | Rodentia | 145 | 28560 |
| neugenii_gene_ensembl | 28 | Mammalia | Diprotodontia | 200 | 15905 |
| ngalili_gene_ensembl | 34 | Mammalia | Rodentia | 145 | 22803 |
| nleucogenys_gene_ensembl | 210 | Mammalia | Primates | 215 | 27529 |
| oanatinus_gene_ensembl | 12 | Mammalia | Monotremata | 200 | 8178.5 |
| oaries_gene_ensembl | 150 | Mammalia | Artiodactyla | 200 | 13733 |
| ocuniculus_gene_ensembl | 31 | Mammalia | Lagomorpha | 200 | 15089 |
| odegus_gene_ensembl | 90 | Mammalia | Rodentia | 145 | 15683 |
| ogarnettii_gene_ensembl | 132.5 | Mammalia | Primates | 215 | 13748.5 |
| olatipes_gene_ensembl | 10 | Actinopterygii | Beloniformes | 135 | 6797.5 |
| oniloticus_gene_ensembl | 7 | Actinopterygii | Perciformes | 135 | 9516 |
| oprinceps_gene_ensembl | 30 | Mammalia | Lagomorpha | 200 | 15466.5 |
| pabelii_gene_ensembl | 259 | Mammalia | Primates | 215 | 17404 |
| paltaica_gene_ensembl | 97.5 | Mammalia | Carnivora | 200 | 22285 |
| panubis_gene_ensembl | 179 | Mammalia | Primates | 215 | 27761 |
| pbairdii_gene_ensembl | 27 | Mammalia | Rodentia | 145 | 20828 |
| pcapensis_gene_ensembl | 240 | Mammalia | Hyracoidea | 200 | 16702.5 |
| pcoquereli_gene_ensembl | 162 | Mammalia | Primates | 215 | 24509 |
| pformosa_gene_ensembl | 30 | Actinopterygii | Cyprinodontiformes | 135 | 10077 |
| pmarinus_gene_ensembl | 5 | Hyperoartia | Petromysontiformes | 65 | 7875 |
| ppaniscus_gene_ensembl | 240 | Mammalia | Primates | 215 | 27893 |
| ppardus_gene_ensembl | 95 | Mammalia | Carnivora | 200 | 22800 |
| psinensis_gene_ensembl | 60 | Reptilia | Testudines | 125 | 19391 |
| ptroglodytes_gene_ensembl | 236 | Mammalia | Primates | 215 | 30083 |
| pvampyrus_gene_ensembl | 180 | Mammalia | Chiroptera | 200 | 13819 |
| rbieti_gene_ensembl | 199 | Mammalia | Primates | 215 | 29680 |
| rnorvegicus_gene_ensembl | 21 | Mammalia | Rodentia | 145 | 17842 |
| rroxellana_gene_ensembl | 199 | Mammalia | Primates | 215 | 29298 |
| saraneus_gene_ensembl | 20 | Mammalia | Eulipotyphla | 200 | 15500 |
| sboliviensis_gene_ensembl | 151.5 | Mammalia | Primates | 215 | 31604 |
| scerevisiae_gene_ensembl | 0.083 | Saccharomycetes | Saccharomycetales | 3 | 1086 |
| sharrisii_gene_ensembl | 21 | Mammalia | Dasyuromorphia | 200 | 17916 |
| sscrofa_gene_ensembl | 114 | Mammalia | Artiodactyla | 200 | 32892.5 |
| tbelangeri_gene_ensembl | 46 | Mammalia | Scandentia | 145 | 19349 |
| tguttata_gene_ensembl | 13.5 | Aves | Passeriformes | 175 | 7654 |
| tnigroviridis_gene_ensembl | 5 | Actinopterygii | Tetraodontiformes | 135 | 3660 |
| trubripes_gene_ensembl | 5 | Actinopterygii | Tetraodontiformes | 135 | 6525 |
| ttruncatus_gene_ensembl | 345 | Mammalia | Catartiodactyla | 200 | 16607 |
| vpacos_gene_ensembl | 345 | Mammalia | Artiodactyla | 200 | 21854.5 |
| xmaculatus_gene_ensembl | 30 | Actinopterygii | Cyprinodontiformes | 135 | 8178 |
| xtropicalis_gene_ensembl | 60 | Amphibia | Anura | 145 | 13237 |
